# Beyond Flory’s principle: Cyclization and unequal reactivity in step-growth linear polymerization

**DOI:** 10.1126/sciadv.adu8884

**Published:** 2025-05-09

**Authors:** Yinghao Li, Hang Yin, Yi Situ, Jing Lyu, Wenxin Wang

**Affiliations:** Charles Institute of Dermatology, School of Medicine, University College Dublin, Dublin 4, Ireland.

## Abstract

Step-growth polymerization (SGP) products are ubiquitous, but the theoretical understanding of SGP reactions has stagnated since the introduction of Flory’s classical theory. Flory’s model based on two key assumptions—equal reactivity of functional groups and the absence of cyclization—falls short in guiding real-world polymerization processes. In this work, we extend Flory’s model by accounting for individual reaction probabilities and cyclization tendencies, making it applicable to real SGP situations. Moreover, we have developed a top-down algorithm capable of extracting crucial information about polymer growth and cyclization from molecular weight data during the SGP process. By applying this expanded model to real SGP experiments, we reveal their kinetic mechanisms and demonstrate how concentration affects polymerization kinetics, offering valuable insights for predicting and controlling the polymer structure.

## Teaser

Flory’s model was refined by including reactivity and cyclization and decodes polymer growth kinetics from molecular weight.

## INTRODUCTION

Step-growth polymerization (SGP) has become one of the most widely used chemical processes for producing polymeric materials. Today, the demand for polymeric materials with well-controlled structures is increasing, driven by the rapid advancements in industries such as automotive, aerospace, building, electronics, medical devices, etc. The advancement of SGP technology depends on a deeper understanding of its kinetics, which is crucial for controlling the synthesis of well-defined polymer materials. Nearly 80 years ago, Flory first established the classical SGP theory, which provides a foundational framework for understanding most SGP systems (*1*, *2*). However, its simplified reaction conditions and neglect of polymerization kinetics limit its applicability in certain systems, where inconsistencies such as a higher dispersity (*Ɖ*) and higher molecular weights (MWs) than predicted by the classical theory (*3*, *4*) and, in some cases, lower *Ɖ* (*5*–*7*) were observed. In addition, although cyclization is known to be favored at low concentrations, this effect is not considered in Flory’s classical theory. Therefore, there is a growing need to refine existing models to account for both ideal and nonideal conditions, thus improving the control of critical polymer structures, such as MW, MW distribution, and cyclization.

Substantial efforts have been made to develop a comprehensive theory for SGP systems. For instance, various modifications have been introduced into Flory’s classical theory based on the differences between ideal and nonideal systems—such as degradation (*8*, *9*), chain exchange (*9*–*12*), chain termination (*13*), cyclization reactions (*14*–*16*), and unequal reactivity of functional groups (*17*, *18*). These include numerical analysis, statistical methods, kinetics, and thermodynamic analysis. However, despite great effort, a universal theory for SGP systems has not yet been established because of two major limitations in previous studies (*5*–*15*): (i) the neglect of differences in kinetic processes, where the reaction kinetics for producing a product with a specific chain length (CL) are assumed to be the same; (ii) typically, only one deviation factor is considered, while all other reaction processes are idealized, preventing the development of a more general theory. Recent studies by De Keer *et al.* (*19*, *20*) proposed a Monte Carlo (MC) simulation system that dynamically adjusts the intermolecular reaction rates during the branched SGP process to explore the effects of cyclization reactions and flow limitations, including viscosity constraints and the influence of CL on diffusion. Unfortunately, this model requires manual input for a range of parameters, such as the intermolecular/intramolecular reaction rate constants, the threshold for allowing intramolecular reactions, the weighting factors for the effects of viscosity and radius of gyration, etc. In practical reactions, because of the wide variety and substantial differences in SGP types and monomer species, these specific parameters are difficult to obtain, limiting the model’s applicability. In this work, we started from linear SGP systems and established an SGP theoretical model that considers both unequal reactivity and cyclization reactions, allowing for the differentiation of reaction step probabilities. By using experimentally accessible kinetic data, such as the relationship between the number-average polymerization degree (${\bar{X}}_{n}$) and the weight-average polymerization degree (${\bar{X}}_{w}$) at different functional group reaction extents, this model provides a more accurate representation of the polymerization behavior in SGP systems under varying kinetic conditions in real experiments, enabling us to better understand how each stage of the polymerization reaction progresses. Consequently, it allows for the prediction and control of polymer structures, including MWs and cyclization, at any given reaction stage.

## RESULTS

### Theoretical treatment

The reaction systems of the SGP of bifunctional monomers can be divided into three types (A and B represent different functional groups, and a and b represent different moles): (i) equimolar A_A + B_B, (ii) nonequimolar A_A + B_B, and (iii) SGP of A_B. Given that the treatment of A_B self-polymerization is the same as that of equimolar A_A + B_B reaction, and the polymerization behavior of nonequimolar A_A + B_B is like the other two reaction systems before reaching the reaction end, the A_B self-polymerization system was taken for the following theoretical derivation.

$$a\;A\_A+a\;B\_B\overset{k}{\to }A\_A\;{\left(B\_\text{BA}\_A\right)}_{a-1}B\_B$$ (1)

$$a\;A\_A+b\;B\_B\overset{k}{\to }A\_A\;{\left(B\_\text{BA}\_A\right)}_{b-1}B\_\text{BA}\_A\;\left(a>b\right)$$ (2)

$$a\;A\_B\overset{k}{\to }A\_B\;{\left(A\_B\right)}_{a-2}A\_B$$ (3)

#### Consideration of unequal reactivity of functional groups

In Flory’s classical theory, there are two main assumptions: (i) The reactivity of all functional groups is equal, and (ii) there are no intramolecular cyclization reactions. However, in some reactions, because of the different positions of functional groups and other factors such as reaction barriers and steric hindrance, the reaction probabilities of each pair of functional groups vary. Therefore, our initial approach is to develop a probabilistic model within Flory’s theoretical framework that can account for variations in the reaction probabilities between different pairs of reactants.

According to Flory’s classical theory, the probability that a chain with length *x* (*x*-nucleotide oligomer) exists when the degree of reaction is P is $P^{x-1}\left(1-P\right)$. However, when the reactivity of each pair of reactants is unequal, the probability differences of forming an *x*-nucleotide oligomer through different reaction pathways should be considered. We introduced a correction factor F(x) to adjust the probability of a chain with length *x* existing: $P^{x-1}\left(1-P\right)F$. Moreover, to form an *x*-nucleotide oligomer, a chain of length *y* needs to react with a chain of length x−y. Because of factors such as energy barriers and steric hindrance, the reactivity of each pair of reactants differs. For example, when forming a 10-nucleotide oligomer, the reactivity of a 2-nucleotide oligomer reacting with an 8-nucleotide oligomer may not be equal to that of two 5-nucleotide oligomers. In addition, it is difficult to identify a general rule for predicting these reactions. Therefore, a correction function Fr(y,x−y) needs to be introduced to represent the difference in reactivity. This function represents the ratio of the actual reactivity between a chain of length y and a chain of length x−y to the ideal state where all functional groups have equal reactivity. Its value has a range of [0,+∞). Next, we will attempt to derive the expression for F(x). The deviation in probability can be understood as the ratio between the chain formation probability when different reaction pathway probabilities exist and the chain formation probability when no pathway differences exist. However, it is also necessary to simultaneously consider the differences in the intrinsic probabilities of the two chains as reaction substrates, which result from the variation in reaction pathways. Therefore, it can be imagined that, because of the influence of the probabilities of the reactants chains x−y and y existing, the expression for F(x) must involve an iterative relationship, and each iteration is necessarily controlled by Fr(y,x−y). On the basis of these relationships, the expression for F(x) can be constructed as follows

$$\begin{array}{c} F\left(x\right)=\\ \frac{F\left(x-1\right)F(1)\text{Fr}\left(x-1,1\right)+F\left(x-2\right)F(2)\text{Fr}\left(x-2,2\right)+\ldots +F\left(1\right)F(x-1)\text{Fr}\left(1,x-1\right)}{F\left(x-1\right)F\left(1\right)+F\left(x-2\right)F\left(2\right)\ldots +F\left(1\right)F\left(x-1\right)},\\ \left(x\geq 2\right) \end{array}$$ (4)

where the numerator represents the deviation in forming chain *x* through different pathways because of reactivity differences, obtained by summing the probabilities of each pathway and the differences in substrate existence probabilities. The denominator represents the deviation in forming chain *x* through different pathways without reactivity differences, obtained by summing the differences in substrate existence probabilities across each pathway.

At this point, the distribution of *x*-nucleotide oligomers can be written as

$$N_{x}=N_{0}P^{x-1}{\left(1-P\right)}^{2}F/\text{Nor}$$ (5)

where *F* represents F(x) in Eq. 4, and Nor is the normalization parameter

$$\text{Nor}=\sum xP^{x-1}{\left(1-P\right)}^{2}F$$ (6)

Then, the molar mass distribution can be known as

$$\frac{W_{x}}{W}=xN_{0}P^{x-1}{\left(1-P\right)}^{2}F/\text{Nor}$$ (7)

Therefore, on the basis of Eqs. 4 to 7, ${\bar{X}}_{n}$ and ${\bar{X}}_{w}$ with the consideration of unequal reactivity of functional groups can be derived

$${\bar{X}}_{n}=\frac{\sum xN_{x}}{\sum N_{x}}=\sum xP^{x-1}\left(1-P\right)F/\text{Nor}$$ (8)

$${\bar{X}}_{w}=\sum x\frac{W_{x}}{W}=\sum x^{2}P^{x-1}{\left(1-P\right)}^{2}F/\text{Nor}$$ (9)

#### Consideration of intramolecular cyclization reactions

Building on the consideration of unequal functional group reactivity, we further examined the effect of intramolecular cyclization reactions. To address this, the functional group consumption for chain growth and the consumption of cyclization need to be distinguished. On the basis of this understanding, the overall degree of reaction P can be divided into $P_{p}$ (for chain propagation) and $P_{c}$ (for the cyclic structure formation in a single reaction). Cyclization is also influenced by various factors and can be approximated as follows: Whenever a chain *x* forms, there is a probability Fc(x) of cyclization occurring. Therefore, when the differences of different functional groups’ reactivities are neglected, at a reaction extent P, the functional group consumption for cyclization is given by

$$P_{c}=\sum \left(1-P\right)P^{x-1}\frac{\text{Fc}}{1+\text{Fc}}$$ (10)

At this point, the functional group consumption for chain propagation is

$$P_{p}=P-P_{c}$$ (11)

The number of chains is

$$N_{p,x}=N{P_{p}}^{x-1}\left(1-P\right)$$ (12)

Also, the number of rings (*x* ≥ 2) is

$$N_{c,x}=N{P_{p}}^{x-1}P_{c}$$ (13)

The number of macromolecules is

$$N=N_{0}\left(1-P+P_{c}\right)=N_{0}\left(1-P_{p}\right)$$ (14)

The distribution of *x*-nucleotide oligomers can be rewritten as

$$N_{x}=N_{0}\left(1-P_{p}\right){P_{p}}^{x-1}\left(1-P\right)+N_{0}\left(1-P_{p}\right){P_{p}}^{x-1}P_{c},\left(x\geq 2\right)$$ (15)

Considering that monomers do not undergo cyclization, ${\bar{X}}_{n}$ and ${\bar{X}}_{w}$ with the consideration of intramolecular cyclization reactions can be obtained

$${\bar{X}}_{n}=\frac{\sum xN_{x}}{\sum N_{x}}=\sum x\left[{P_{p}}^{x-1}\left(1-P\right)+{P_{p}}^{x-1}P_{c}\right]-P_{c}$$ (16)

$$\begin{array}{c} {\bar{X}}_{w}=\sum x\frac{W_{x}}{W}=\frac{\sum x^{2}N_{x}}{\sum {\mathrm{xN}}_{x}}\\ =\sum x^{2}\left[\left(1-P_{p}\right){P_{p}}^{x-1}\left(1-P\right)+\left(1-P_{p}\right){P_{p}}^{x-1}P_{c}\right]-\left(1-P_{p}\right)P_{c} \end{array}$$ (17)

Furthermore, on the basis of Eqs. 16 and 17, combined with Eqs. 8 and 9, we can derive expressions for ${\bar{X}}_{n}$ and ${\bar{X}}_{w}$ that simultaneously account for both unequal reactivity and cyclization reactions, as shown below

$$\begin{array}{c} {\bar{X}}_{n}=\frac{\sum xN_{x}}{\sum N_{x}}\\ =\sum x\left[{P_{p}}^{x-1}\left(1-P\right)+{P_{p}}^{x-1}P_{c}\right]F/\text{Nor}-P_{c}F/\text{Nor} \end{array}$$ (18)

$$\begin{array}{c} {\bar{X}}_{w}=\sum x\frac{W_{x}}{W}=\frac{\sum x^{2}N_{x}}{\sum {\mathrm{xN}}_{x}}\\ =\sum x^{2}\left[{\left(1-P_{p}\right)}^{2}{P_{p}}^{x-1}F/\text{Nor}\right]-\left(1-P_{p}\right)P_{c} \end{array}$$ (19)

**Fig. 1.**
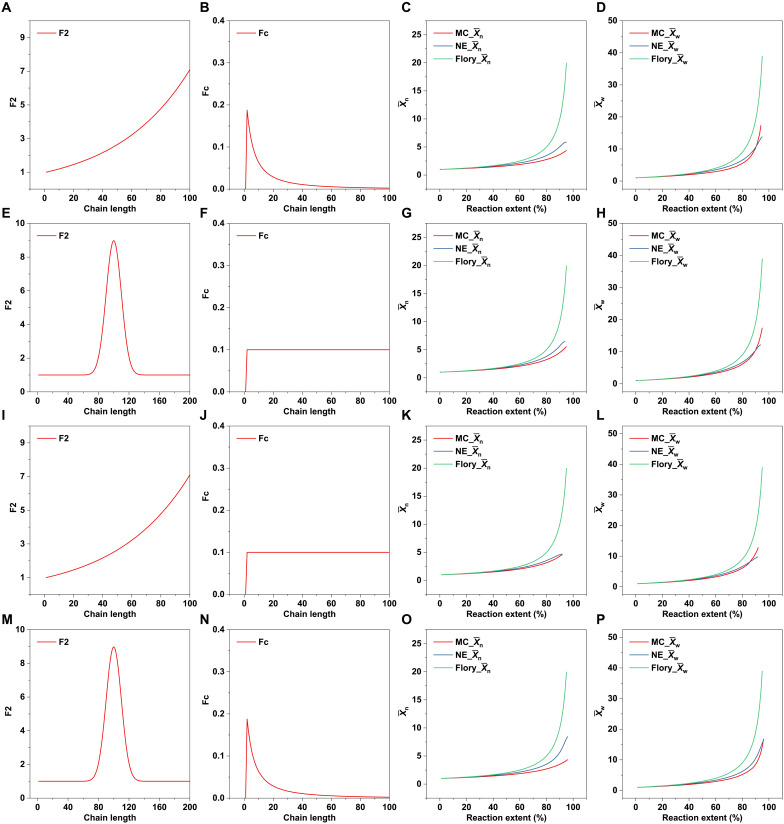
Polymerization behavior of linear SGP predicted by different models (NE, MC, and Flory) at different reactivity and different cyclization trends. (i) Evolution of (**A**) ${\bar{X}}_{n}$ and (**B**) ${\bar{X}}_{w}$ in a system where reactivity increased with CL (**C**) and the cyclization trend first increased and then decreased with CL (**D**). (ii) Evolution of (**E**) ${\bar{X}}_{n}$ and (**F**) ${\bar{X}}_{w}$ in a system where reactivity first increased and then decreased with CL (**G**) and the cyclization trend is constant for different chains (**H**). (iii) Evolution of (**I**) ${\bar{X}}_{n}$ and (**J**) ${\bar{X}}_{w}$ in a system where reactivity increased with CL (**K**) and the cyclization trend is constant (**L**). (iv) Evolution of (**M**) ${\bar{X}}_{n}$ and (**N**) ${\bar{X}}_{w}$ in a system where reactivity first increased and then decreased with CL (**O**) and the cyclization trend first increased and then decreased with CL (**P**). NE calculations were based on Eqs. 18 and 19. To facilitate assigning values to Fr(y,x−y), F2(x) was introduced and its functional expression is constructed as follows: Fr(y,x−y)=F2(y)F2(x−y), F2(1)=1, Fr(1)=0 (see the detailed algorithm description in Materials and Methods).

The validity of the formula was verified by MC simulations under four extreme conditions: (i) The cyclization tendency increases initially and then decreases with increasing CL, while functional group reactivity increases (Fig. 1, A to D); (ii) the cyclization tendency remains constant, while reactivity first increases and then decreases with increasing CL (Fig. 1, E to H); (iii) the cyclization tendency remains constant, while reactivity increases with increasing CL (Fig. 1, I to L); and (iv) the cyclization tendency increases initially and then decreases with increasing CL, while reactivity first increases and then decreases with increasing CL (Fig. 1, M to P). Concatenation (*21*) may occur in experiments, particularly in high-viscosity systems at the late stages of the reaction. However, because of the low probability of this occurrence, this phenomenon is not considered in this model. It can be seen that the results from the new equation (NE) model (Eqs. 18 and 19), when compared with MC simulations, show that, in all cases, the ${\bar{X}}_{w}$ matches well, while ${\bar{X}}_{n}$ exhibits slight deviation. Although the NE model does not perfectly match, compared to the Flory classical model, it still provides a much-improved approximate expression that offers a quantitative theoretical model for predicting and analyzing MWs in complex systems.

### Studying polymerization kinetics using the formula

In real polymerizations, the differences in cyclization reactions and the reactivity of different molecular chains affect the polymerization process. These differences cause various polymerization systems to deviate to varying degrees from Flory’s ideal conditions. If this information can be obtained, it would allow a complete understanding of every step of the polymerization reaction and enable the prediction and control of the structure and composition of polymers at any given reaction point. Therefore, on the basis of the expression for ${\bar{X}}_{n}$ and ${\bar{X}}_{w}$ established in this work (Eqs. 18 and 19), we further explored how to reverse engineer to understand the reactivity and cyclization tendencies of different polymer chains from known polymerization kinetics data. By comparing known experimental results (${\bar{X}}_{n}$ and ${\bar{X}}_{w}$ at different reaction stages) and continuously adjusting parameters via computer simulations, a theoretical curve that matches the experimental data can be obtained. The parameters at this point will reveal the cyclization reactions and reactivity of different molecular chains in the polymerization system.

**Fig. 2.**
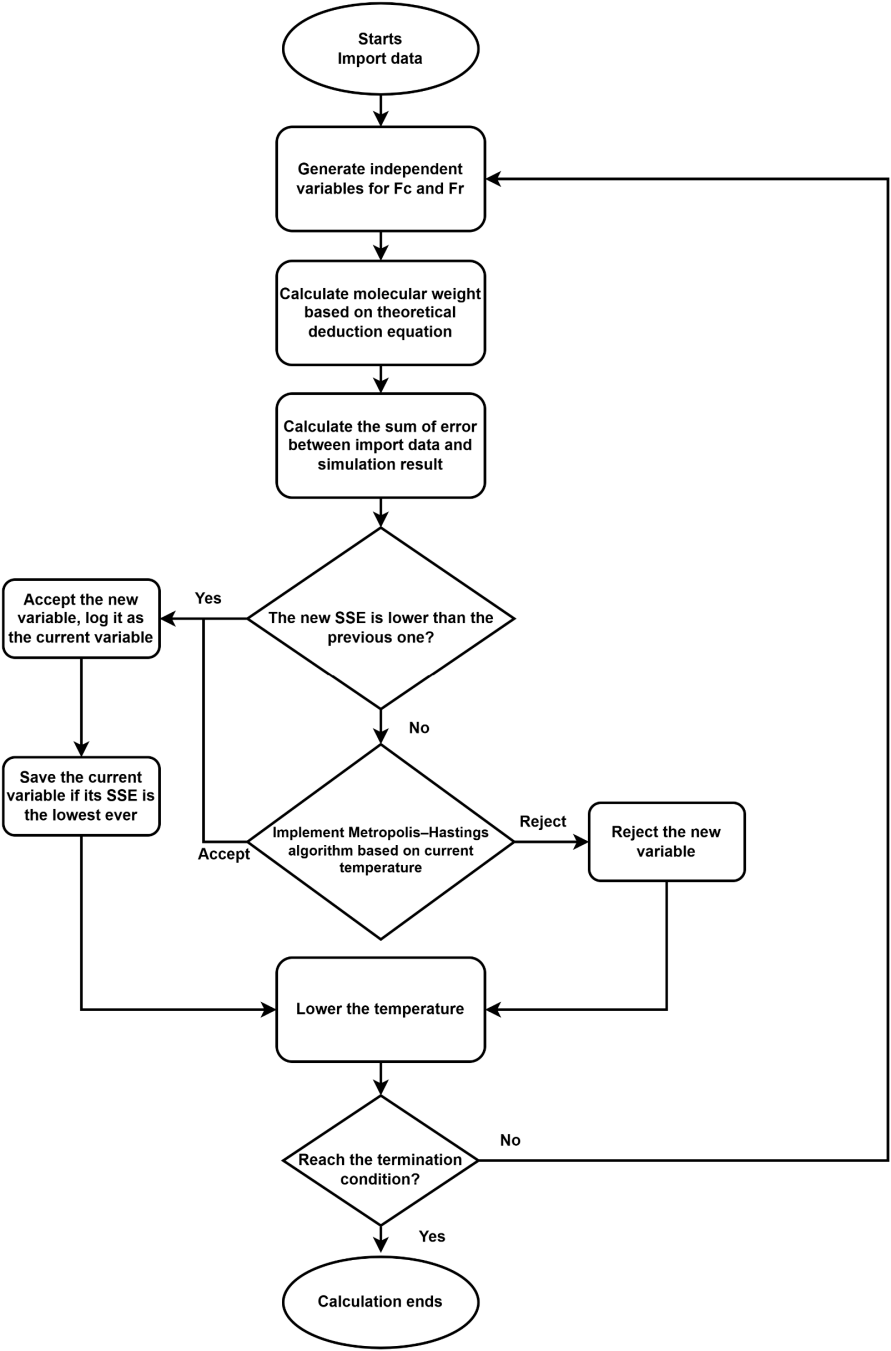
Flowchart of the annealing algorithm for searching the reactivity and cyclization tendency of chains with different lengths.

A simulated annealing algorithm (Fig. 2) was developed to identify specific values for Fr(y,x−y) and Fc(x) based on the ${\bar{X}}_{n}-P$ and ${\bar{X}}_{w}-P$ curves, allowing us to explore the reactivity and cyclization tendencies across chains with different lengths. The known ${\bar{X}}_{n}-P$ and ${\bar{X}}_{w}-P$ curves (which can be obtained through experiments) will be input into the algorithm. The algorithm will randomly set a series of values for Fr(y,x−y) and Fc(x) and use these values to calculate the ${\bar{X}}_{n}$ and ${\bar{X}}_{w}$ curves according to Eqs. 18 and 19.

The calculated results will then be compared with the imported ${\bar{X}}_{n}$ and ${\bar{X}}_{w}$ curves to compute the error based on the sum of squared errors (SSE). The Metropolis criterion is then used to decide whether to accept the new variables while gradually lowering the temperature. This process is repeated until the SSE is reduced to a minimal value. At this point, the optimized values of Fr(y,x−y) and Fc(x) can be obtained from the algorithm.

Because the values of Fr(y,x−y) and Fc(x) are directly related to polymerization kinetics, they can be used to establish a complete kinetic process applicable to the current reaction system. With this information, combined with MC simulations, it is possible to quickly and accurately achieve any necessary predictions, thereby enabling precise control over the polymer structure, MW, degree of cyclization or any other structural parameter of the target polymer.

**Fig. 3.**
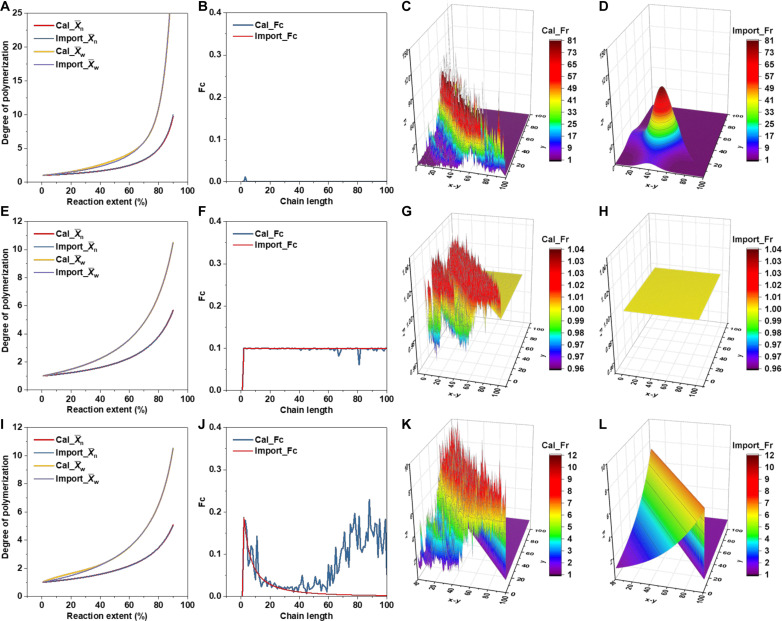
Determining reactivity and cyclization trends of chains with different lengths in Flory’s ideal system using the annealing algorithm. Three systems under extreme conditions were evaluated: (i) (**A** to **D**) a system with consistent functional group reactivity but differing cyclization tendencies, (ii) (**E** to **H**) a system devoid of cyclization but exhibiting varying reactivities, and (iii) (**I** to **L**) a complex system incorporating both cyclization reactions and unequal functional group reactivities. [(A), (E), and (I)] ${\bar{X}}_{n}$ and ${\bar{X}}_{w}$ calculated from algorithm simulation (Cal) and input data from origin system (Import). [(B), (F), and (J)] Values of Fc(x) calculated from algorithm simulation (Cal_Fc) and values from origin system (Import_Fc). [(C), (G), and (K)] Simulated values of Fr(y,x−y) from the origin system. [(D), (H), and (L)] Origin values of Fr(y,x−y) from the origin system, which work as a comparison to the results of (C), (G), and (K). The import systems of (A) to (D) are the same as those in fig. S2 (G to I). The import systems of (E) to (H) are the same as those in fig. S3 (D to F). The import systems of (I) to (L) are the same as those in Fig. 1 (A to D).

To verify the applicability of the algorithm, we applied several ${\bar{X}}_{n}-P$ and ${\bar{X}}_{w}-P$ curves derived from known parameters [Fr(y,x−y) and Fc(x)] to the algorithm. If the parameters provided by the algorithm correspond to the original parameters, it would demonstrate the feasibility and reliability of this method. Three systems with extreme conditions were tested: (i) a system with the same functional group reactivity but varying cyclization tendencies, (ii) a system without cyclization but with different reactivities, and (iii) a complex system involving both cyclization reactions and unequal functional group reactivities. The values of Fr(y,x−y) and Fc(x) were determined (Fig. 3). Although, in Fig. 3J, the Cal_Fc is noisy at CL > 40, this is because the cyclization of the long chain has little effect on ${\bar{X}}_{n}$ and ${\bar{X}}_{w}$ in this system. This causes the algorithm to fall to a local optimum, but it does not affect the global result. Overall, the combination of this algorithm with the formulas derived in this work is successful, providing an excellent tool for the study of polymerization kinetics.

**Fig. 4.**
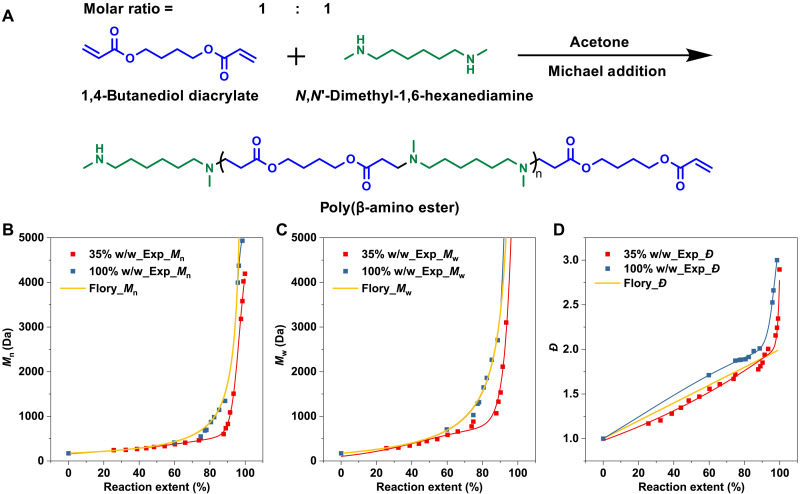
Polymerization behaviors of PAE under different reaction concentrations. (**A**) Schematic diagram of the structure of the monomer and PAE. Evolution of (**B**) $M_{n}$, (**C**) $M_{w}$, and (**D**) *Đ* under different reaction concentration systems. The experimental $M_{n}$ and $M_{w}$ were obtained from GPC, and the reaction extent was obtained from nuclear magnetic resonance spectroscopy (tables S1 and S2).

After verifying the applicability of the algorithm, we applied it to the analysis of real polymerization experiments. Equimolar “A_2_ + B_2_” poly(β-amino ester) (PAE) SGP experiments based on the Michael addition reactions were performed at two different concentrations (100 and 35%, w/w) (Fig. 4A). Briefly, experiments were carried out with equimolar 1,4-butanediol diacrylate and *N*,*N*′-dimethyl-1,6-hexanediamine in acetone. From Fig. 4 (B and C), it can be observed that, before the reaction reaches completion (reaction extent <90%), at high concentration (100%, w/w), the curves for number-average MW ($M_{n}$) and weight-average MW ($M_{w}$) are very close to Flory’s predicted results. However, near the end of the reaction, the experimental $M_{w}$ value increases rapidly, and the *Đ* exceeds the theoretical limit of 2.0 (Fig. 4D) predicated by Flory’s classical theory. In contrast, in the low-concentration (35%, w/w) reaction, both $M_{n}$ and $M_{w}$ are lower than Flory’s predictions and the high-concentration system, which does demonstrate the effect of reaction concentration. Then, toward the end of the reaction, the $M_{w}$ value also increases rapidly, causing *Đ* to exceed 2.0, although it remains lower than the results from the high-concentration system at the same reaction extent.

**Fig. 5.**
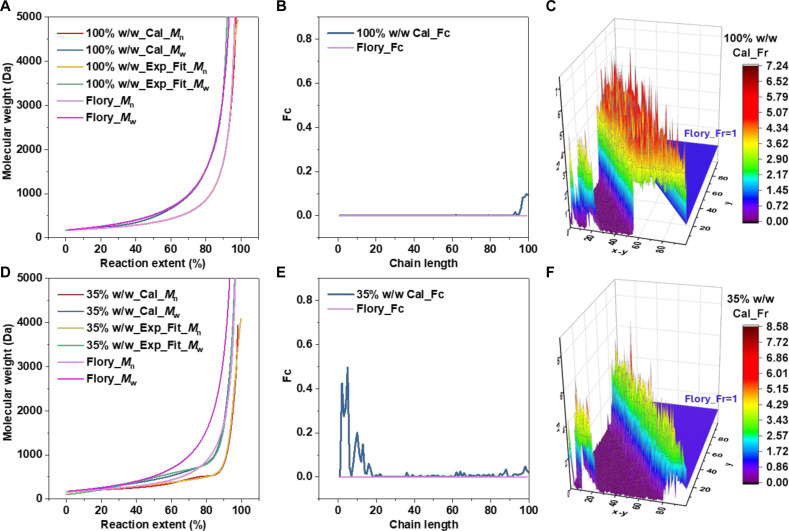
Determining reactivity and cyclization trends of chains with different lengths in a PAE SGP system using the annealing algorithm. (**A** and **D**) $M_{n}$ and $M_{w}$ from algorithm simulation (Cal) and input data (Import). (**B** and **E**) Values of Fc(x) from algorithm simulation (Cal). (**C** and **F**) Simulated values of Fr(y,x−y) from the origin system.

On the basis of the above experimental results, we applied the $\text{Exp}\_M_{n}-P$ and $\text{Exp}\_M_{w}-P$ curves to the algorithm to study the cyclization [Fc(x)] and reactivity [Fr(y,x−y)] properties of chains with different lengths during the reaction. The results show that, for both 35 and 100% (w/w) concentrations, the algorithm-fitted $\text{Cal}\_M_{n}-P$ and $\text{Cal}\_M_{w}-P$ curves closely match the original curves, indicating that the kinetic results from the experiments were matched (Fig. 5, A and D). The analysis results of Fc(x) show that, at a high concentration of 100% (w/w), almost no cyclization reactions occur. Cyclization only occurs slightly when CL exceeds 90, while by this point, the reaction is nearing completion and the system’s viscosity is high. The reactive chain ends of different molecular chains have difficulty coming into contact, which increases the chance of intramolecular reactions. In contrast, for polymers that react at a low concentration of 35% (w/w), the cyclization trend initially increases rapidly with CL and then gradually decreases to zero. The cyclization trend peaks around a CL of 5 (MW of 855 Da) and almost no cyclization occurs for chains longer than 20 (MW of 3420 Da) (Fig. 5, B and E).

For a more intuitive understanding, fig. S4 presents curves with the *x* axis represented in actual length (Å) or the number of bonds. Conversely, the analysis results of Fr(y,x−y) show that, for chains with lengths less than 50, the reactivity is quite similar, regardless of whether the reaction concentration is high (100%, w/w) or low (35%, w/w). However, the reaction concentration affects the formation of long chains. At high concentrations (100%, w/w), the reactivity for forming long chains (CL > 50) increases rapidly. In contrast, for low-concentration reactions (35%, w/w), this increase in reactivity is delayed until the formation of chains is longer than 80, and the degree of increase is lower (Fig. 5, C and F). This is because short-chain formation typically occurs in the early stages of the reaction, where system viscosity is lower, making molecular movement and conformation changes easier. Long-chain formation generally occurs at the later stages of the reaction, accompanied by a rapid increase in system viscosity. As a result, adjacent long chains are more likely to react compared to distant short chains. This explains why *Đ* increases rapidly at the end of the reaction, deviating from Flory’s theoretical limit (2.0).

The analysis results are consistent with expectations: The reactivity of chains is determined by their chemical structure and is less influenced by reaction concentration in the early stages. Intramolecular reactions forming rings occur more readily at low concentrations, while cyclization becomes increasingly difficult as CL increases. This work provides a more intuitive understanding: High reaction concentrations promote the formation of long chains at the end of the reaction; chains with MWs below 3420 Da are more affected by reaction concentration and are more likely to undergo cyclization at low concentrations.

The prediction results are very positive, as they allow us to understand how each pair of molecules reacts during the polymerization process. By comparing different experimental conditions (with reaction concentration as an example in this study), we can study how reaction conditions affect the reactivity of each pair of reactants and the cyclization trend of different chains, ultimately influencing the polymerization process. With this information about Fc(x) and Fr(y,x−y), the polymerization of PAE under 100 and 35% (w/w) conditions can be accurately predicted. Notably, although the model established in this work can predict equimolar linear SGP systems under either ideal or nonideal conditions, it still has limitations in explaining more complex SGPs (such as A*_x_* + B*_y_*, A_2_ + B_3_ + C_4_, etc.). This is due to the more complex distribution of molecular chains in such systems and the fact that functional groups on the same molecule in branched polymers may exhibit different reaction probabilities because of steric hindrance. These aspects will be thoroughly studied and discussed in our next work.

## DISCUSSION

To improve Flory’s framework, we considered the reactivity and cyclization tendency of each pair of functional group reactions, leading to the construction of an approximate expression for ${\bar{X}}_{n}$ and ${\bar{X}}_{w}$. This approach offers valuable insights for the theoretical study of SGP reactions. Moreover, an algorithm was developed on the basis of the derived formulas, enabling a bottom-up analysis of the reactivity and cyclization behavior of chains of different lengths within the reaction system from experimentally obtainable ${\bar{X}}_{n}$ and ${\bar{X}}_{w}$ results. The analytical findings help us understand the progression of each step in the polymerization reaction, allowing for precise prediction and control of the polymer structure throughout the process. We further validated the expanded Flory theory by studying the reaction kinetics of an SGP system at various concentrations. This model, under different realistic SGP initial parameter sets, elucidated how each step of a polymerization reaction occurs and how it is influenced by concentration. We anticipate that this expanded framework will be applied to a wider range of polymerization systems, offering valuable insights into their reaction kinetics and how different conditions affect their polymerization behavior.

## MATERIALS AND METHODS

### Materials

1,4-Butanediol diacrylate and *N*,*N*′-dimethyl-1,6-hexanediamine were purchased from Sigma-Aldrich. Lithium bromide (LiBr) for gel permeation chromatography (GPC) measurements was purchased from Sigma-Aldrich. Acetone, dimethylformamide (DMF), and diethyl ether were purchased from Thermo Fisher Scientific. Deuterated chloroform (CDCl_3_) was purchased from Sigma-Aldrich.

### Experimental model selection

To verify the applicability of the new theory model, experiments were carried out with equimolar 1,4-butanediol diacrylate and *N*,*N*′-dimethyl-1,6-hexanediamine. In this case, diacrylates copolymerize with amines via facile Michael addition, representing a typical A_2_ + B_2_ reaction.

The PAE is a newly developed biological material, which is widely used in gene therapy and drug delivery (*22*, *23*). Although attempts have been undertaken to decipher the relationship between its structure and performance, the precise control of polymer topology is rarely mentioned, whereas most of these monographs still focused on bioapplications. Moreover, the short-range effect and configuration influence are neglected because of the soft alkyl interval. Therefore, this PAE polymer is an essential and ideal model for the investigation of size-length–dependent inequality and concentration effects in SGP.

### Polymer synthesis

PAEs were synthesized through a Michael addition reaction. For the PAE synthesized at 35% (w/w), equimolar 1,4-butanediol diacrylate (3.96 g) and *N*,*N*′-dimethyl-1,6-hexanediamine (2.88 g) were dissolved in 15.96 ml of acetone in a 50-ml two-necked flask. Then, the reactions were carried out in an ice bath (0°C).

For the PAE synthesized at 100% (w/w), equimolar 1,4-butanediol diacrylate (3.96 g) and *N*,*N*′-dimethyl-1,6-hexanediamine (2.88 g) were put together without a solvent. Then, the reactions were carried out in the ice bath.

### Polymer characterization

An Agilent 1260 Infinite GPC equipped with a triple detector [a refractive index detector (RI), a viscometer detector (VS DP), and a dual light scattering detector (LS 15° and LS 90°)] was used to monitor the change of functional group conversion rate, *M*_w_, *M*_n_, and *Ɖ*. For GPC measurement, 20 μl of the reaction mixture was taken and diluted in 1 ml of DMF and filtered through a 0.45-μm filter. DMF with 0.1% LiBr was used to elute the GPC columns (Polar Gel-M, 7.5 by 300 mm, two in series) at a flow rate of 1 ml/min at 60°C. Linear poly(methyl methacrylate) standards were used for the calibration of the GPC columns. Meanwhile, the conversion rates and chemical compositions were monitored with ^1^H nuclear magnetic resonance spectroscopy on a 400-MHz Varian Inova spectrometer. The samples were reported in parts per million (ppm) relative to the solvent CDCl_3_ (7.23 ppm) or internal control (tetramethylsilane; 0.00 ppm).

### MC algorithm description

In the traditional Flory ideal system, reactions between functional groups are considered to have equal reactivity, meaning that they occur with equal probability, and no intramolecular reactions occur. In the work by De Keer *et al.* (*19*), they introduced additional functions to adjust the intermolecular reaction rate constants, allowing them to change with the extent of the reaction. This adjustment altered the competition between intermolecular and intramolecular reactions—specifically, chain growth and cyclization reactions. By this method, they simulated the effects of increasing system viscosity as the reaction progressed, which restricted chain mobility and ultimately reduced the rate of intermolecular reactions.

In this MC algorithm, we directly adjusted the probability differences for each pair of reactions. A correction factor Fr(y,x−y) is introduced to adjust the probability weight of each reaction pair (a chain of length *y* and a chain of length *x-y*). The values of Fr(y,x−y) are preset, ranging from 0 to infinity. The Flory system, where all reaction reactivities are equal, is used as a reference frame, with all Fr(y,x−y) values set to 1. In addition, in the Flory system, cyclization is not allowed. However, in this simulation system, each chain has a probability of undergoing cyclization, depending on its CL, represented by Fc(x), after it is formed. The value of Fc(x) is also preset at the beginning. In the Flory system, all Fc(x) values are 1. Because of computational limitations, the cutoff value for *x* in the preset Fr(y,x−y) and Fc(x) values is set to 400, meaning that the longest allowed chain in the system is 400 units. Our validation confirmed that this CL is sufficient to cover all simulations. The flow chart of MC is shown in fig. S1. Below are some examples of reactions:

For the Flory system in fig. S2 (A to C), all Fr(y,x−y) values are 1, and all Fc(x) values are 0. In this case, every pair of functional groups has an equal probability of being selected for reaction.

For the system with unequal functional group reactivity but no cyclization in fig. S2 (D to F), all Fc(x) values are 0. Considering that the value of Fr(y,x−y) is affected by both the chain of length y and the chain of length x−y, the expression of Fr(y,x−y) was constructed to reflect the joint effects of chain y and chain x−y. Fr(y,x−y) values were set using another two-dimensional function F2, where Fr(y,x−y)=F2(y)F2(x−y), referring to the reaction between a chain of length y and a chain of length x−y. F2 is only used for Fr assignment and has no actual physical meaning. This method simplifies the assignment of the Fr(y,x−y) matrix. In this case, the probability of each functional group pair being selected for reaction is no longer equal but is adjusted on the basis of the value of Fr(y,x−y) before being randomly selected.

For the system with both unequal functional group reactivity and cyclization in Fig. 1 (A to D), both Fr(y,x−y) and Fc(x) values are preset. After adjusting the reaction probabilities based on Fr(y,x−y), two chains are randomly selected for reaction, and then cyclization is determined on the basis of the probability set by Fc(x). An example of the core structure of the MC program is provided in the Supplementary Materials, written in the R programming language.

### Results comparison between MC and formulas

The Eqs. 8 and 9 were first validated in the ideal system with equal reactivity (fig. S2, A to C). The complete overlap of the Flory model, MC, and the NL in both ${\bar{X}}_{n}$ and ${\bar{X}}_{w}$ proves the reliability of the NL. Subsequently, in the second case, the reactivity was set to increase exponentially with CL in one case (fig. S2, D to F) and, in the third one, to increase first and then decrease (fig. S2, G to I). The results show that, while the MC results deviate notably from the classical Flory curve, they align well with the NL results, demonstrating the applicability of the NL to the unequal reactivity polymerization condition.

We then compared the cases where the functional group has equal reactivity, but the chain cyclization tendency was different (Eqs. 16 and 17). As shown in fig. S3, when the functional group reactivity is equal and no cyclization occurs [all Fr(y,x−y) values are set as 1, and Fc(x) values are set as 0], the Flory theoretical curve, computer simulation curve, and the newly derived formula overlap perfectly (fig. S3, A to C). Subsequently, the cases where functional group reactivity is equal but cyclization tendencies differ were studied. Two scenarios were considered: one where the cyclization tendency remains constant as CL increases (fig. S3, D to F) and another where it first increases and then decreases (fig. S3, G to I). The results show that, while the computer simulation results deviate significantly from the classical Flory curve, they align well with the new formula for the ${\bar{X}}_{w}$. However, there is still some deviation for the ${\bar{X}}_{n}$ to MC results.

### Simulated annealing algorithm description

The logic of this program revolves around the fitting and optimization of MW data, with the workflow proceeding as follows: The program first loads experimental data, which contain *M*_n_ and *M*_w_ under different reaction extents. The experimental data are then fitted using PCHIP interpolation to get the $M_{n}-P$ and $M_{w}-P$ curves as targets. The program calculates the required values of Fr(y,x−y) and Fc(x) using random number generation and then uses these values to compute $M_{n}-P$ and $M_{w}-P$ curves based on the newly derived formulas. Subsequently, the program evaluates the difference between the theoretical values and the experimental data by calculating the total error (SSE). This error serves as the optimization criterion, with the fitting performance for the $M_{n}-P$ and $M_{w}-P$ curves being assessed individually. A simulated annealing algorithm is used as the optimization technique in this process, aiming to minimize the loss function. Through gradual temperature reduction and random perturbations, the algorithm searches for the best-fit parameters that minimize the SSE, resulting in the optimal $M_{n}-P$ and $M_{w}-P$ curves. Last, the program outputs the computed values of Fr(y,x−y) and Fc(x), which were used to generate the MW curves, providing insight into the fitting process and the theoretical model’s behavior.

## Supplementary Materials

### This PDF file includes:

Monte Carlo Algorithm Code Figs. S1 to S4 Tables S1 and S2
