# Supplementary material for: Beyond Flory’s principle: Cyclization and unequal reactivity in step-growth linear polymerization
Source: Sci Adv. 2025 May 9;11(19):eadu8884. doi: 10.1126/sciadv.adu8884 (PMC12063651; doi:10.1126/sciadv.adu8884)
Supplement: Supplementary file 1 — Monte Carlo Algorithm Code Figs. S1 to S4 Tables S1 and S2 [file sciadv.adu8884_sm.pdf]

Science Advances
AAAS

# Supplementary Materials for

## Beyond Flory's principle: Cyclization and unequal reactivity in step-growth linear polymerization

Yinghao Li *et al.*

Corresponding author: Jing Lyu, jing.lyu@ucd.ie; Wenxin Wang, wenxin.wang@ucd.ie

*Sci. Adv.* **11**, eadu8884 (2025)
DOI: 10.1126/sciadv.adu8884

**This PDF file includes:**

Monte Carlo Algorithm Code
Figs. S1 to S4
Tables S1 and S2

***Monte Carlo Algorithm Code***

An example of the core structure of the MC program is provided below, written in the R programming language:

```
### Basic Settings
rm(list = ls())
options(max.print = 20)

# Simulation parameters
Calculate_length <- 1000      # Maximum chain length to calculate
P <- seq(0, 0.93, by = 0.01) # Extent of reaction
Size <- 30000                 # Number of simulation particles
cyclization <- TRUE           # Include cyclization (TRUE/FALSE)

# Cyclization probabilities for different chain lengths
cyclization_prob <- dlnorm(1:(Calculate_length * 10) / 10, meanlog = 0, sdlog = sqrt(5 / 2)) / 4
cyclization_prob[1] <- 0      # Cyclization probability for chain length 1 is zero

# Weighting for reaction probabilities
k_speed <- 1.0
wlr <- numeric(10000)
wlr[1] <- 1
for (i in 2:10000) {
  wlr[i] <- k_speed * wlr[i - 1]
}

### Functions

# Number average degree of polymerization (DP)
X_n_bar <- function(Sys) {
  Sys$Initial / ((length(Sys$Particle) + length(Sys$Cycle)) / 2)
}

# Weight average DP
X_w_bar <- function(Sys) {
  sum(c(Sys$Particle[, 1], Sys$Cycle[, 1])^2) / Sys$Initial
}

# Perform one Monte Carlo reaction step
MCstep_react <- function(Sys, cyc, prob) {
  Particle <- Sys$Particle
  len <- nrow(Particle)
  done <- FALSE

  while (!done) {
```

```
    index <- sort(sample(len, 2, replace = FALSE, prob = wlr[Particle[, 1]]))
    if (Particle[index[1], 2] != Particle[index[2], 2] || (Particle[index[1], 2] == 0 &&
Particle[index[2], 2] == 0)) {
      temp <- Particle[index[1], ] + Particle[index[2], ]

      if (cyc && temp[2] == 0 && runif(1) < prob[temp[1]]) {
        Sys$Cycle <- rbind(Sys$Cycle, temp)
        Particle <- Particle[-index, , drop = FALSE]
      } else {
        Particle <- Particle[-index[2], , drop = FALSE]
        Particle[index[1], ] <- temp
      }

      Sys$Particle <- Particle
      len <- nrow(Particle)
      cyc.n <- nrow(Sys$Cycle)
      Sys$P <- c(1 - len / Sys$Initial, 0, cyc.n / Sys$Initial)
      Sys$P[2] <- Sys$P[1] - Sys$P[3]
      done <- TRUE
    }
  }
  return(Sys)
}

# Visualize simulation results
Drawnow <- function(P, Sys) {
  par(mfrow = c(1, 3), mar = c(6, 4, 6, 2))

  # Number average DP plot
  plot(P, Sys$X_n, xlab = "P", ylab = expression(bar(X[n])), main = expression(bar(X[n])), type
= "l")
  lines(P, 1 / (1 - P), col = "red")
  legend("topleft", c("Monte Carlo", "Flory"), col = c("black", "red"), lty = 1, cex = 0.8)

  # Weight average DP plot
  plot(P, Sys$X_w, xlab = "P", ylab = expression(bar(X[w])), main = expression(bar(X[w])),
type = "l")
  lines(P, (1 + P) / (1 - P), col = "red")
  legend("topleft", c("Monte Carlo", "Flory"), col = c("black", "red"), lty = 1, cex = 0.8)

  # PDI plot
  plot(P, Sys$PDI, xlab = "P", ylab = expression(PDI), main = expression(PDI), type = "l")
  lines(P, (1 + P) / ((1 - P)^2), col = "red")
  legend("topleft", c("Monte Carlo", "Flory"), col = c("black", "red"), lty = 1, cex = 0.8)
}
```

```r
### Main Simulation

# Initialize the system
System <- list(
  Initial = Size,
  Particle = matrix(c(rep(1, Size), rep(0, Size)), Size, 2),
  X_n = c(1, rep(NA, length(P) - 1)),
  X_w = c(1, rep(NA, length(P) - 1)),
  PDI = c(1, rep(NA, length(P) - 1)),
  Cycle = matrix(NA, 0, 2),
  P = c(0, 0, 0)
)
colnames(System$Particle) <- c("Chain_length", "Type")

# Progress bar for simulation
pb <- txtProgressBar(min = 1, max = length(P), style = 3)
for (i in 2:length(P)) {
  while (System$P[1] < P[i]) {
    System <- MCstep_react(System, cyclization, cyclization_prob)
  }

  System$X_n[i] <- X_n_bar(System)
  System$X_w[i] <- X_w_bar(System)
  System$PDI[i] <- System$X_w[i] / System$X_n[i]
  setTxtProgressBar(pb, i)
}
close(pb)

# Display results
Drawnow(P, System)
```

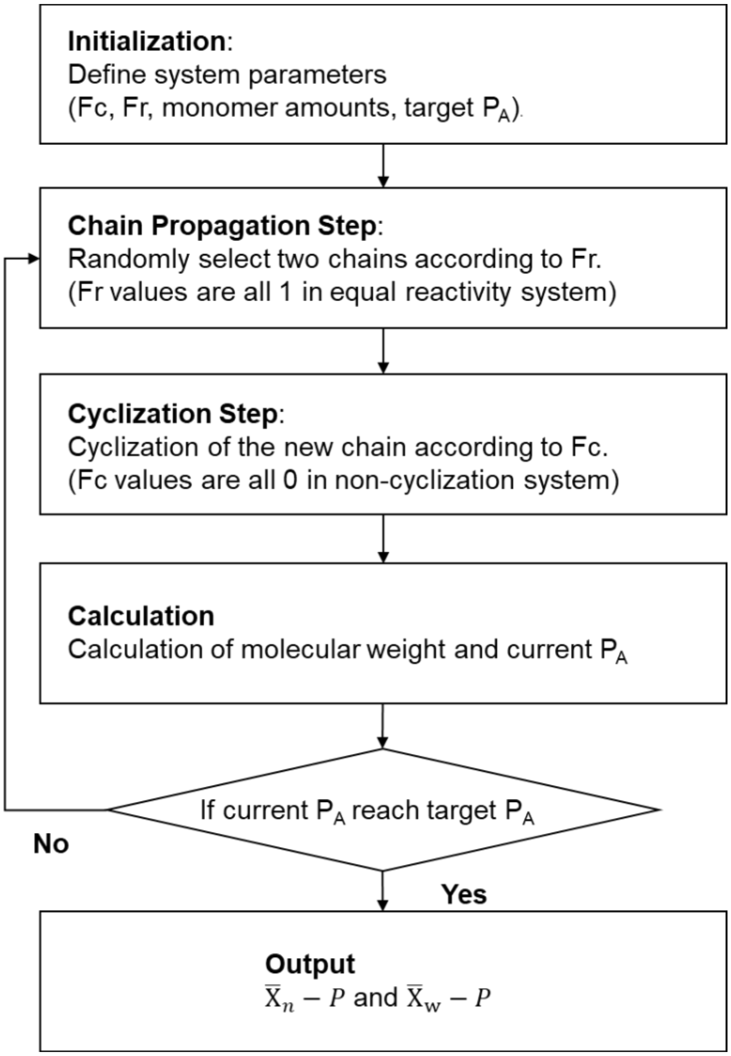


**Fig. S1.** Flowchart of Monte Carlo simulation.

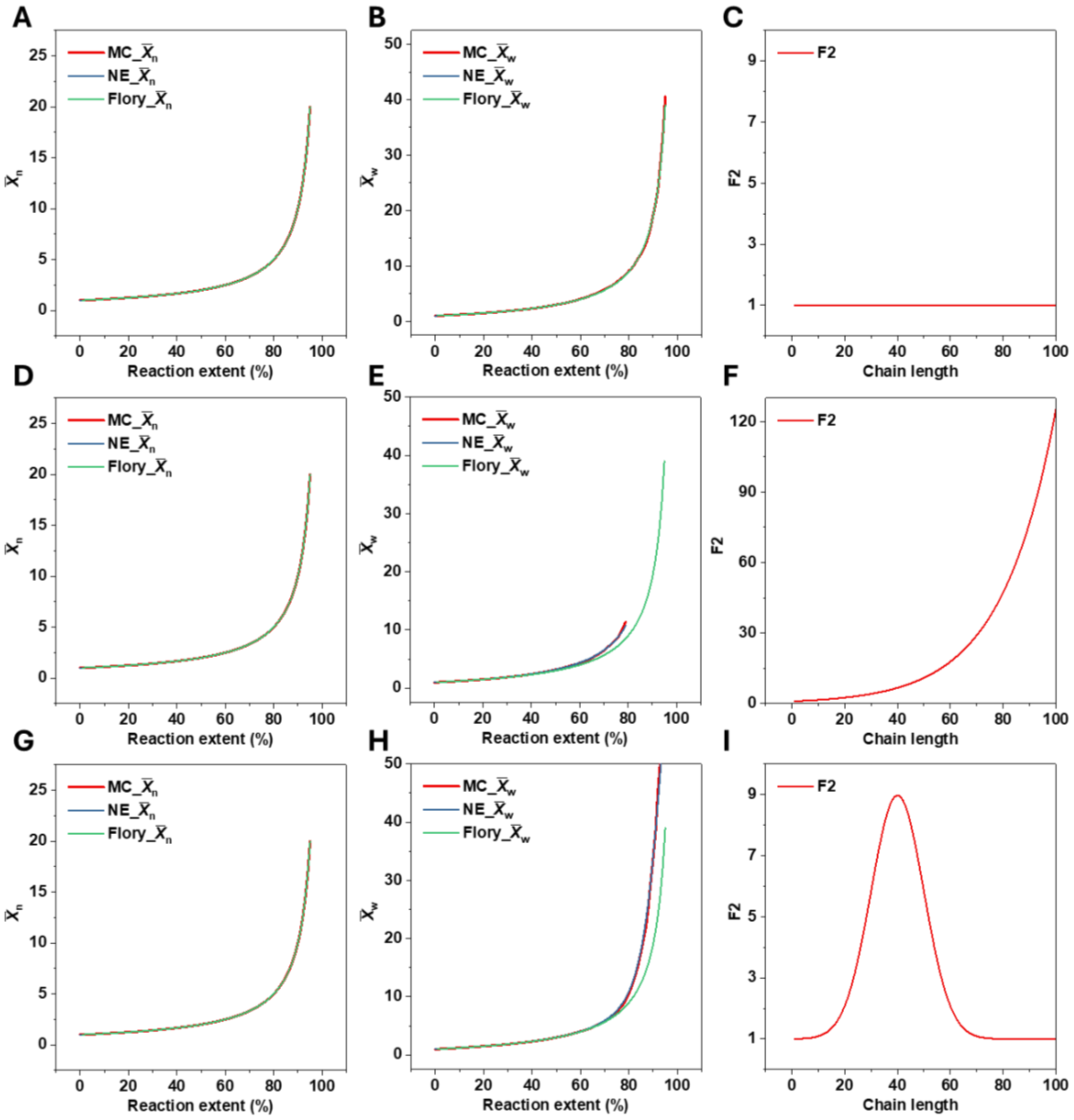


**Fig. S2.** The polymerization behavior of linear SGP predicted by the Flory classical model, MC computer experiments, and the new equation (NE) at different functional group reactivity-chain length relationship. Evolution of the (**A**) $\bar{X}_n$ and (**B**) $\bar{X}_w$ under Flory ideal system. The reactivity of chains with different length were equal, so all values of F2 were set as 1 (**C**). Evolution of the (**D**) $\bar{X}_n$ and (**E**) $\bar{X}_w$ in a system where reactivity increased with chain length (**F**). Evolution of the (**G**) $\bar{X}_n$ and (**H**) $\bar{X}_w$ in a system where reactivity first increased then decreased with chain length (**I**). Calculations were based on Equations (5), (6). $F2(1) = 1$, $Fr(1) = 0$.

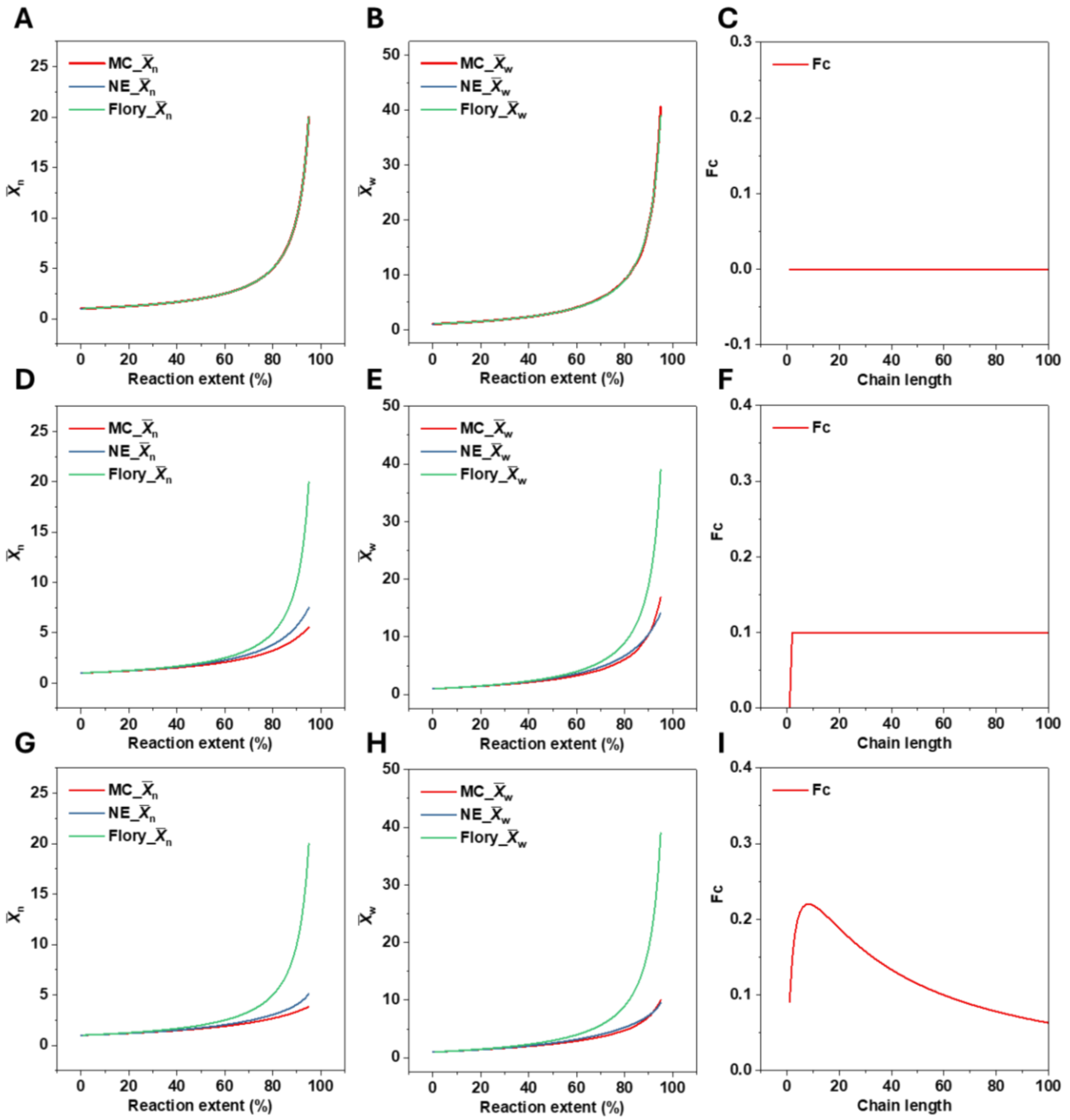


**Fig. S3.** The polymerization behavior of linear SGP predicted by different models at different cyclization trends. Evolution of the (**A**) $\bar{X}_n$ and (**B**) $\bar{X}_w$ under Flory ideal system. There is no cyclization during the polymerization, so the values of Fr were set as 0 (**C**). Evolution of the (**D**) $\bar{X}_n$ and (**E**) $\bar{X}_w$ in a system where cyclization trend is constant for different chains (**F**). Evolution of the (**G**) $\bar{X}_n$ and (**H**) $\bar{X}_w$ in a system where cyclization trend first increased then decreased with chain length (**I**). Calculations were based on Equations (15), (16). $F2(1) = 1$, $Fr(1) = 0$.

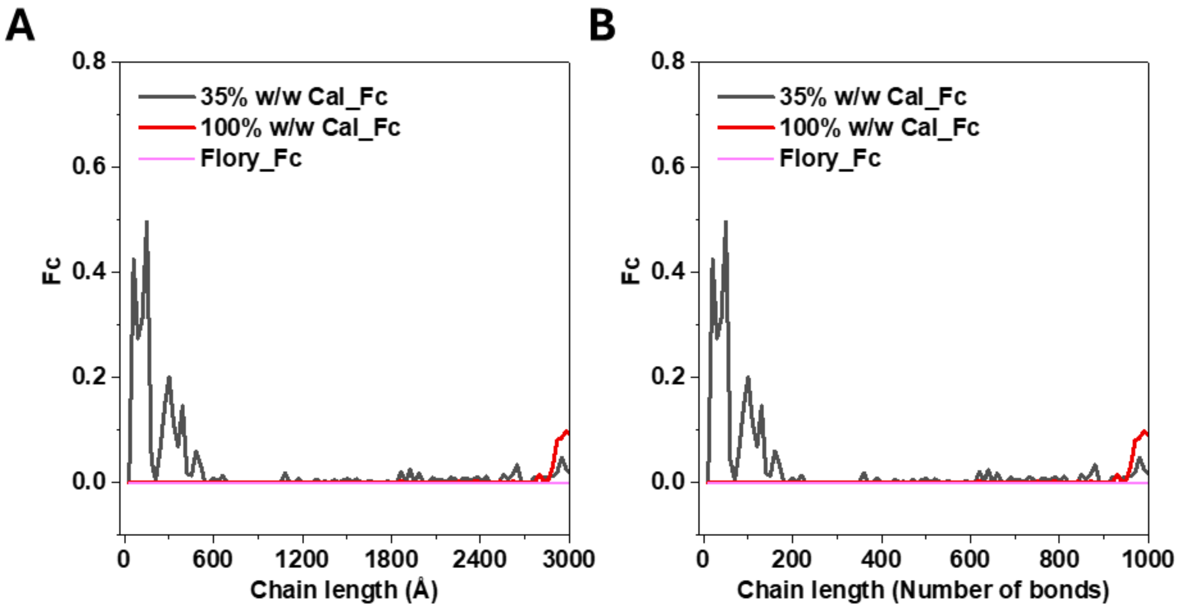


**Fig. S4.** Values of $Fc(x)$ from algorithm simulation (Cal). (A) The *x*-axis represents the actual length. (B) The *x*-axis represents the number of (C-C/C-N/C-O) bonds. One repeating unit includes 6 C-C bonds, 2 C-O bonds, and 2 C-N bonds. The length of a single C-C bond is approximately 3.08 Å, a C-O bond is approximately 2.86 Å, and a C-N bond is approximately 2.94 Å. Therefore, the length of one repeating unit is approximately 30.08 Å. Bond lengths were measured from the chain geometry optimized using Gaussian 16W with the B3LYP density functional theory method and the 6-31+G(d,p) basis set, which includes diffuse functions (+) and polarization functions (d, p).

**Table S1.** Evolution of the PAE (35% w/w)

| **$M_n$ (Da)** | **$M_w$ (Da)** | ***Đ*** | **Reaction extent (%)** |
|---|---|---|---|
| 171 | 171 | 1 | 0 |
| 244 | 286 | 1.17 | 25.6 |
| 249 | 300 | 1.205 | 32.4 |
| 270 | 346 | 1.281 | 38.8 |
| 288 | 388 | 1.347 | 44.0 |
| 313 | 446 | 1.427 | 48.4 |
| 333 | 490 | 1.471 | 54.4 |
| 376 | 586 | 1.559 | 60.2 |
| 410 | 660 | 1.610 | 66.0 |
| 464 | 775 | 1.670 | 73.8 |
| 513 | 882 | 1.719 | 74.8 |
| 602 | 1070 | 1.777 | 87.8 |
| 734 | 1329 | 1.811 | 89.0 |
| 829 | 1535 | 1.852 | 90.2 |
| 1089 | 2113 | 1.940 | 91.4 |
| 1508 | 3103 | 2.005 | 93.4 |
| 3183 | 6863 | 2.156 | 97.6 |
| 3582 | 8037 | 2.243 | 98.4 |
| 4025 | 9443 | 2.346 | 99.0 |
| 4195 | 11242 | 2.895 | 99.8 |

**Table S2.** Evolution of the PAE (100% w/w)

| $M_n$ (Da) | $M_w$ (Da) | *Đ* | Reaction extent (%) |
|---|---|---|---|
| 171 | 171 | 1 | 0 |
| 413 | 707 | 1.712 | 59.8 |
| 549 | 1029 | 1.874 | 74.8 |
| 682 | 1283 | 1.881 | 77.2 |
| 698 | 1316 | 1.885 | 78.0 |
| 872 | 1648 | 1.890 | 80.6 |
| 982 | 1861 | 1.915 | 82.4 |
| 1145 | 2268 | 1.981 | 85.2 |
| 1346 | 2707 | 2.011 | 88.6 |
| 3997 | 10097 | 2.526 | 95.8 |
| 4375 | 11646 | 2.661 | 96.4 |
| 4934 | 14825 | 3.000 | 98.4 |
